# Kangaroo Care on High-Frequency Jet Ventilation: Overcoming Perceived Barriers in Micro Preemies with Birth Weights Less than 750 Grams

**DOI:** 10.3390/children13020310

**Published:** 2026-02-23

**Authors:** Aparna Patra, Pratibha Thakkar, Lisa D. McGee, Prasad Bhandary, Peter J. Giannone, Elie G. Abu Jawdeh

**Affiliations:** 1 Pediatrix Neonatology of Texas, Pediatrix Medical Group, Fort Worth, TX 76104, USA; 2 Division of Neonatology, Department of Pediatrics, University of Kentucky, Lexington, KY 40508, USA; pratibha-thakkar@ou.edu (P.T.);; 3 Nursing Practice and Support Services, University of Kentucky, Lexington, KY 40508, USA; 4 Division of Neonatology, Department of Pediatrics, University of Texas Southwestern Medical Center and Children’s Medical Center, Dallas, TX 75390, USA

**Keywords:** kangaroo care, developmental care, jet ventilator, preterm infants, hypoxemia, temperature instability

## Abstract

Objective: Kangaroo care (KC) is underutilized in preterm infants on ventilator support due to perceived physiologic instability. The objective of our study is to demonstrate the feasibility of safe KC provision on high-frequency jet ventilation (HFJV) in micro preemies weighing less than 750 g. Study Design: Our neonatal intensive care unit has a multidisciplinary clinical standard for KC while preterm infants are on HFJV (HFJV-KC). Bedside staff documented cardiorespiratory and physiologic parameters pre, during, and post HFJV-KC. We performed a retrospective assessment of the feasibility of HFJV-KC in the micro preemie population. Results: A total of 96 HFJV-KC occurrences from 13 neonates with median gestational age 24 1/7 weeks and birth weight of 670 g were included. There were no significant differences in heart rate and temperature pre, during, and post-HFJV-KC. There were statistically significant improvements in oxygen saturation and fraction of inspired oxygen post HFJV-KC. Secondary analyses of prolonged HFJV-KC beyond 1 h (mean 2.3 h) compared to the standard 1 h duration also showed no differences in outcome measures. Conclusions: This study demonstrates that KC may be performed in the smallest micro preemie infants (<750 g) on HFJV. Our study also presents processes to overcome perceived barriers of HFJV-KC implementation in a vulnerable population.

## 1. Introduction

Kangaroo care (KC) is the practice of skin-to-skin contact between a naked diaper-clad infant and the bare chest of a parent. Benefits of KC are well described in the neonatal literature for the parent-infant dyad and include a multitude of physiologic, behavioral, and long-term psychosocial benefits for both babies and parents. Infants undergoing KC show reduced fluctuations in cardiorespiratory parameters, maintain thermal homeostasis, better autonomic responses to painful procedures, experience enhanced weight gain, demonstrate more mature sleeping patterns leading to better sleep organization and have higher cognitive scores at long-term follow up [1,2,3,4,5]. Parental benefits include emotional aid during physical separation from the infant leading to stress reduction, improved maternal mood, more productive breast feeding and allowing practice of family integrated care for infants admitted to the neonatal intensive care unit (NICU) [6,7]. As evidence has emerged in favor of practicing KC, it has been endorsed as standard of care in the NICU by organizations such as World Health Organization, American Academy of Pediatrics and American Academy of Breast Feeding Medicine [8,9].

Despite these well recognized benefits and published guidelines for provision of KC in NICU patients [10,11,12], this practice remains underutilized in many NICUs specifically in the extremely premature population on invasive respiratory support [13]. Barriers to this valuable care are primarily related to NICU culture and perceived difficulty in provision of KC for ‘unstable’ preterm infants who may benefit from KC experiences [14]. Although there is rising evidence of feasibility and safety of KC in infants on ventilator support, there is paucity of studies in the smallest extremely preterm infants on high-frequency jet ventilator (HFJV) [15]. The objective of our study is to demonstrate the feasibility of safe KC provision on HFJV to extremely premature infants less than 750 g. This study described detailed procedures for safe provision of KC to the most preterm infants on HFJV, which is increasingly used in NICUs for managing respiratory failure as survival at lower gestation continues to improve.

## 2. Materials and Methods

The NICU of the University of Kentucky in Lexington, KY, is a Level IV regional referral center. HFJV had been established as the primary intention mode of ventilation for the management of acute respiratory failure in extremely preterm infants as part of routine clinical care in this NICU. Given the perceived difficulties of KC on HFJV, we formed a multidisciplinary quality improvement group of neonatologists, nurses, and respiratory therapists to develop a KC clinical practice guideline for premature infants < 750 g on HFJV. Educational material in the form of protocol and video using a mannequin showing the detailed method of transfer of infant on HFJV was developed and disseminated amongst NICU staff for their learning and practice (https://chs.uky.edu/physician-assistant-studies/resources/kangaroo-care, accessed on 10 January 2026). In this study, we included infants with gestation ≤ 28 weeks and birth weight < 750 g who received KC on HFJV (HFJV-KC). Bedside staff documented vital signs pre, during, and post HFJV-KC. Data was obtained retrospectively from electronic medical records. The study was reviewed and approved by the Institutional Review Board of the University of Kentucky as a quality improvement initiative and granted a waiver of informed consent.

**Figure 1:**
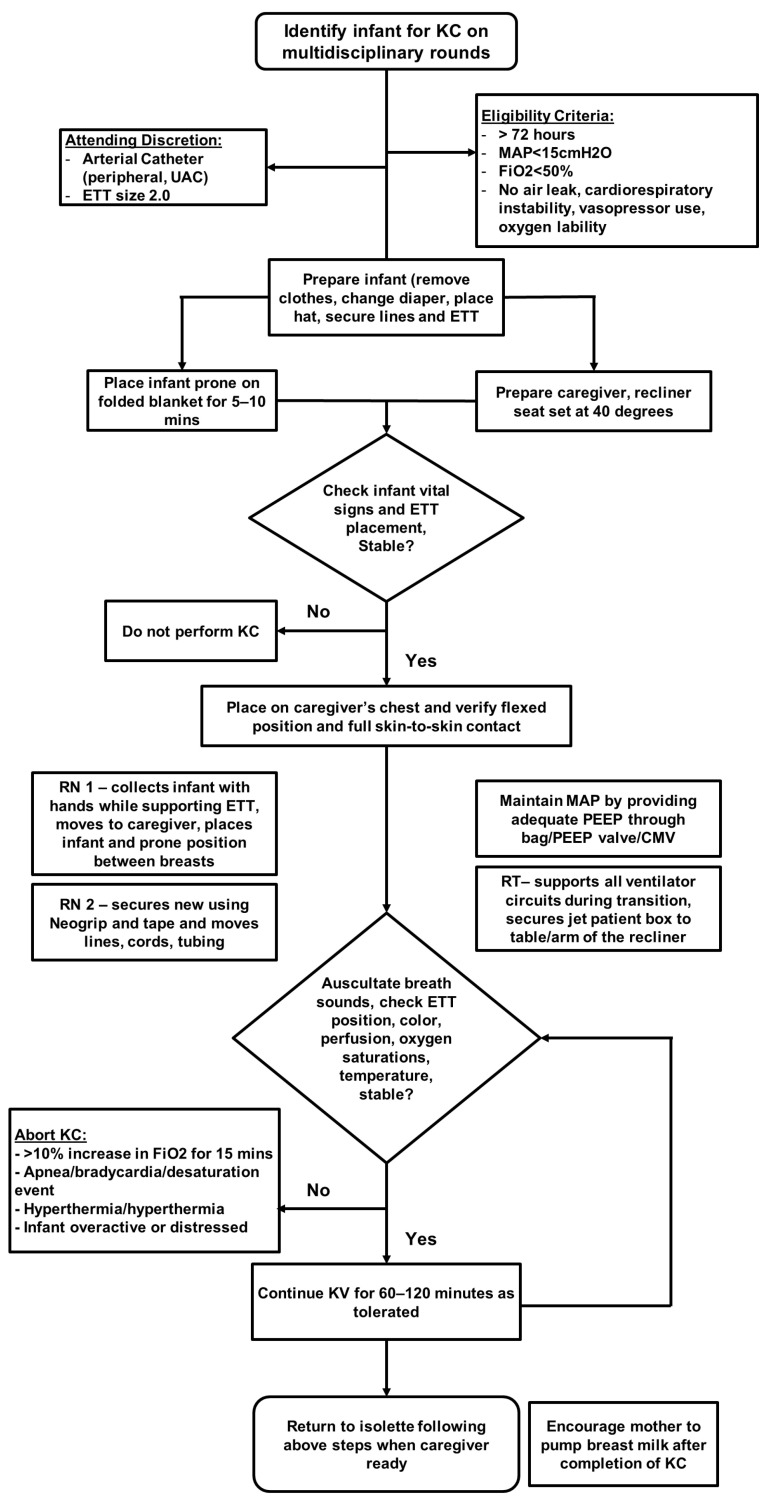
Clinical practice guideline for performance of kangaroo care while on jet ventilator.

The provision of KC to the study subjects was in concordance with the unit protocol on HFJV-KC and approval by the attending neonatologist. The detailed method for transfer of an infant on HFJV from incubator to parent’s chest and back to incubator is illustrated in Figure 1. Our transfer method utilizes two nurses and two respiratory therapists. Special consideration is given to safe transfer/positioning of the infant: keeping the complex dual ventilator circuit of HFJV intact, no loss or decrease in baseline positive end expiratory pressure during transfer and ensuring non-dislodgement of endotracheal tube.

All extremely premature infants on HFJV greater than 72 h old, on HFJV mean airway pressure < 15 mm of Hg, fractional inspired oxygen < 0.5, without oxygen lability were deemed suitable for HFJV-KC after approval by the attending neonatologist. Presence of air leak and hemodynamic instability requiring vasopressor support were considered to be exclusion criteria. At the attending neonatologist’s discretion, the presence of an arterial catheter or use of a 2.0 endotracheal tube did not preclude participation. HFJV-KC was offered to families during each parental visit when the infant met protocol criteria and a caregiver was available, with sessions performed for a minimum of 60 min. The time criteria used was based on established unit protocol and literature [12] to avail the maximum benefits without causing alterations in the infant’s resting state. Parents continued with longer duration of HFJV-KC provided the infant demonstrated clinical stability throughout the period as frequently assessed by nursing staff.

During HFJV-KC, the bedside nurse ensured physiologic stability by monitoring the optimal infant prone kangaroo position, ensuring patency of airway, and checking that tubes and cords were properly connected to the ventilator circuit and monitor. Infants remained on continuous cardiorespiratory and pulse oximetry monitoring during HFJV-KC and transfers (Intellivue MX800, Philips Healthcare, Andover, MA, USA). In addition to continuous electronic monitoring, physiologic parameters were checked by bedside nurse every 30 min for infants undergoing HFJV-KC per protocol to evaluate clinical stability. Physiologic parameters were assessed at three time intervals: (1) Pre HFJV-KC (neonate in incubator), (2) during HFJV-KC (30 min after neonate placed on parent’s chest) and (3) post HFJV-KC (neonate returned to incubator). For neonates who received HFJV-KC for longer than 1 h, multiple recordings of physiologic parameters were taken during HFJV-KC depending on duration of HFJV-KC; the first recording (taken 30 min after initiation of HFJV-KC) was used for statistical purposes. Data was extracted on heart rate (HR), oxygen saturation (SpO_2_), respiratory rate, axillary temperature (T) and fractional inspired oxygen (FiO_2_) on HFJV. All cardiorespiratory parameters were automatically imported from Phillips Intellivue monitor to vital sign recordings in electronic medical records. With HFJV, high-velocity gas pulses are delivered at higher frequency, making it difficult to reliably assess the patient’s spontaneous breathing rate. Hence, change in respiratory rate was not included in the analysis. Information on adverse events during or after HFJV-KC, such as inadvertent or accidental endotracheal tube dislodgement or escalation in respiratory support was also obtained. All neonates in our study received standard dose of oral or intravenous caffeine 10 mg/kg/day. Neonates in our study cohort continued to receive orogastric tube feeds during HFJV-KC.

Descriptive statistics were used to summarize the baseline clinical characteristics of our study cohort including gestational age, birthweight, day of life (DOL) for first hold and cumulative duration of KC per patient (hours). We applied paired sample *t*-test or non-parametric tests to compare changes in physiological parameters temperature, heart rate and oxygen saturations baseline values before initiation of HFJV-KC, during HFJV-KC and after completion of HFJV-KC. We examined the data to evaluate if alterations in physiologic parameters were observed with longer duration of HFJV-KC performance > 1 h. IBM SPSS Statistics version 27 and GraphPad Prism version 10 were used for statistical analysis.

## 3. Results

There were 96 total HFJV-KC occurrences from 13 extremely preterm infants with birth weight ≤ 750 g during the study period. Of those, 3, 3 and 12 HFJV-KC occurrences were excluded during analyses of HR, SpO_2_ and FiO_2_ analyses respectively due to missing cardiorespiratory data in electronic medical records. We compared temperature changes pre and post HFJV-KC only; temperature datapoints recorded during HFJV-KC were excluded due to low documentation rate. Neonatal characteristics of the study group are summarized in Table 1. Our study exclusively included neonates born < 750 g on HFJV with a median gestational age of 24.1 weeks [Interquartile range (IQR) 23 4/7–24 6/7 weeks] and a median birth weight of 670 g (IQR 603–708 g). The median time to first hold was 16 days (IQR 13–20 days). The median cumulative duration of skin-to-skin contact per neonate in our cohort was 11 h (interquartile range, 6–15 h). The rate of parental readiness to participate in KC was 44% (96 times HFJV-KC performed out of 218 times offered). 

**Figure 2:**
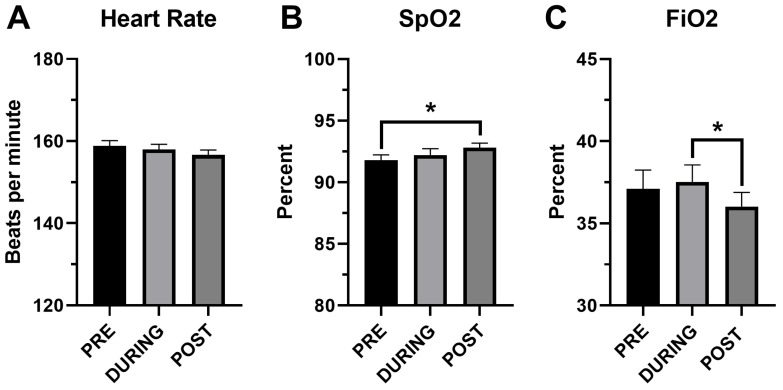
Comparison of cardiorespiratory variables before, during and after HFJV-KC. (**A**) Heart rate, n = 93. (**B**) SpO_2_—oxygen saturation by pulse oximetry, n = 93. (**C**) FiO_2_—fractional inspired oxygen on HFJV, n = 84. PRE—Before initiation of HFJV-KC (infant in incubator). DURING—30 min into performance of HFJV-KC. POST—after completion of HFJV-KC (infant returned to incubator). * *p* < 0.05.

**Figure 3:**
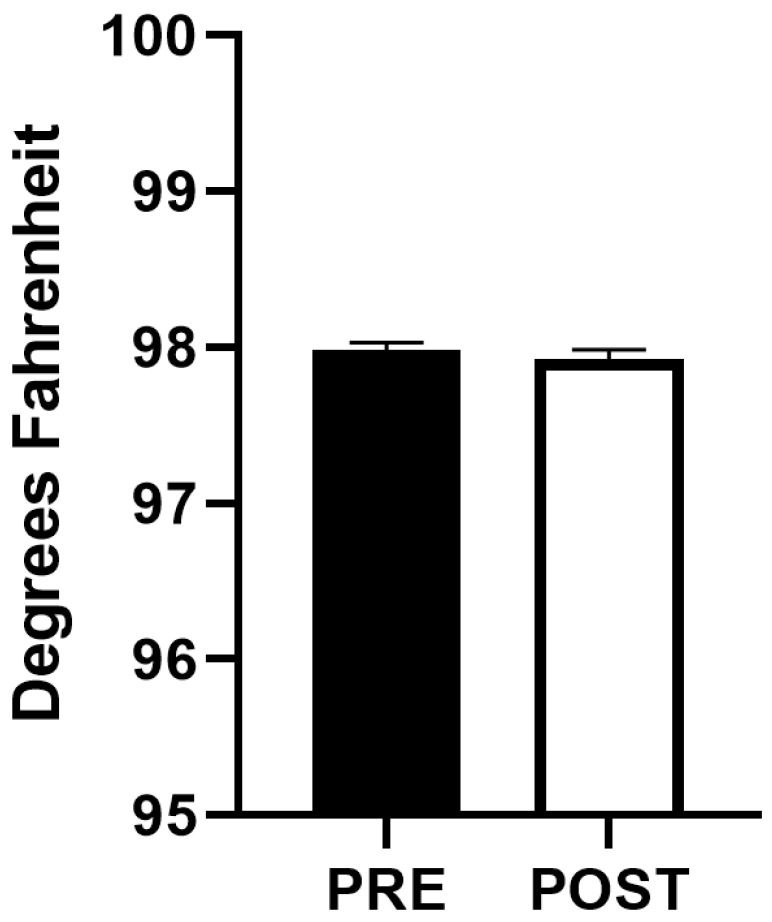
Comparison of axillary temperature before initiation and after completion of HFJV-KC (n = 88). PRE—Before initiation of HFJV-KC (infant in incubator). POST—after completion of HFJV-KC (infant returned to incubator).

Cardiorespiratory parameters before, during and after performance of HFJV-KC are compared in Figure 2. Comparison of T pre and post HFJV-KC are presented in Figure 3. As noted in the figures, there were no statistically significant differences in HR (158.8 ± 1.3 vs. 158 ± 1.2 vs. 156.6 ± 1.2, mean ± SEM, *p* = 0.29) in the three time points pre, during and post HFJV-KC, respectively. Increase in oxygen saturation noted post HFJV-KC (92.8 ± 0.4) compared to pre HFJV-KC (91.8 ± 0.4), though statistically significant (mean ± SEM, *p* = 0.046), is likely not clinically relevant. A decrease in FiO_2_ requirement noted in post HFJV-KC (36.02 ± 0.9) compared to during HFJV-KC (37.5 ±1.03) was statistically significant (Mean ± SEM, *p* = 0.002). No statistically significant changes were observed in other FiO_2_ comparisons. No change in temperature (98 ± 0.04 vs. 97.9 ± 0.05, mean ± SEM, *p* = 0.21) was observed pre and post HFJV-KC.

To study the effect of prolonged HFJV-KC on infant stability, we divided the events into two groups: those with standard KC duration of 1 h (mean 1.0 h) compared to KC > 1 h (mean 2.3 h). There were no statistically significant differences in HR, SpO_2_, FiO_2_ and T while comparing infants in the 1 h vs. >1 h groups as noted in Table 2.

We did not observe significant bradycardia (HR < 80 bpm) or oxygen desaturation events requiring FiO_2_ increment of >10% from baseline for >15 min which would result in early termination of HFJV-KC followed by return to incubator. There were 2/96 occurrences of inadvertent dislodgement of endotracheal tube in our study cohort during HFJV-KC. This video illustrates performance of HFJV-KC by parents of extremely premature babies on HFJV at our center.

## 4. Discussion

Our study of provision of KC on HFJV focuses on the most vulnerable patient population of extremely premature babies with birth weight less than 750 g. This study presents processes to overcome perceived barriers of implementation of HFJV-KC in this specific patient population. Secondly, our findings demonstrate that KC in infants on HFJV is safe when a team approach is utilized, and infants are monitored closely. Feasibility and safety of kangaroo care for premature babies on invasive and noninvasive respiratory support has been an area of inquiry for many researchers [12,15,16,17,18] as the advantages of this care are well recognized in the premature population [4,5,15,19,20,21]. Most of these prior studies have demonstrated physiologic stability in premature neonates of broader gestational age and higher birth weight ranges. The fear of physiologic instability during transfer of a neonate on high-frequency ventilation has precluded its widespread adoption in NICUs. Our findings expand the evidence provided by other investigators and further establishes that skin-to-skin care of extremely premature micro preemie (<750 g) intubated with complicated jet ventilator circuit and equipment is achievable and secure if standardized guidelines are followed by a trained team of NICU staff. The standardized processes presented in this manuscript may help units safely implement HFJV-KC on their most premature patients.

The physiological parameters studied in our report were similar and unchanged between pre HFJV-KC and post HFJV-KC. We noted statistically significant improvement in oxygen saturations on completion of HFJV-KC, which is reassuring; however, the difference may not be clinically significant. Our findings are comparable to other studies including preterm patient cohorts [15,16,17,18]. Furthermore, our population is comprised of a homogeneous group of smaller and more immature neonates on invasive high-frequency ventilation. Prior studies have included neonates on both invasive and noninvasive respiratory support which may have led to hesitancy in translation of the positive results to the smallest preterm infant. Lorenz et al. [17] also demonstrated stability in cerebral oximetry readings monitored by near-infrared spectroscopy in addition to physiologic parameters both during transfer of infant as well as during skin-to-skin care. Although we did not document physiologic data during transfer, our transfer protocol ensures calibration of positive end expiratory pressure (PEEP) valve and providing equivalent positive end expiratory pressure to the ventilator, thus preventing potential loss of mean airway pressure leading to desaturations or respiratory instability during the transfer process.

All HFJV-KC occurrences in this study continued for at least 60 min per our standard guidelines. In fact, the majority (66/96 = 69%) of our HFJV-KC occurrences continued for >1 h. There were no differences in physiologic parameters between pre and post HFJV-KC for occurrences of 1 h duration compared to >1 h duration; this is an important finding that may allow prolonged HFJV-KC in the future to optimize benefits to the parent–infant dyad.

Since neonates in our cohort were continuously on a jet ventilator, we were unable to analyze their spontaneous respiratory rate. Periodic apnea previously described [22,23] during kangaroo care in premature babies could not be assessed while the neonate was on HFJV in our study. Our nursing staff ensured appropriate prone positioning and airway patency to optimize neonatal lung mechanics [24,25], leading to decreased incidence of hypoxemic events during kangaroo care [18].

Our incidence of inadvertent endotracheal tube dislodgement during HFJV-KC was comparable to other non-kangaroo care-related causes of unplanned extubations (such as loose endotracheal tube securement, repositioning of baby, nursing care, emesis, agitation/spontaneous movement in baby) in the same study period for all extremely premature neonates on HFJV in the NICU. To address concerns regarding baseline risk, we previously examined unplanned extubation rates among extremely premature infants on HFJV who were not receiving kangaroo care compared to HFJV-KC. These rates were similar, and provision of HFJV-KC was not associated with an increased risk of unplanned extubation [26]. This finding further supports implementing HFJV-KC in this highly vulnerable population.

A limitation of our study is the small sample size due to our focus on a homogeneous group of extremely preterm infants < 750 g and the retrospective nature of the study. Given that this is a single center study, our results may not be generalizable to every NICU. We have skilled champion nurses who primarily take care of this target population of patients. Their keen attention to detail of invasive lines and tubes, compliance with clinical protocol and expertise in transfer of patient with a complicated jet ventilator circuit may be crucial to the physiologic stability noted in our study. To facilitate implementation of KC in the micro preemie population, we share our HFJV-KC transfer method and an educational video in this manuscript. We also noted that in our patient cohort, parental readiness to perform HFJV-KC was 44%, which underlines the need for continued parental guidance through visual aids and videos of HFJV-KC performance and educating families about the benefits of skin-to-skin care in the smallest premature neonates.

In conclusion, as survival rates of the smallest preterm infants steadily increase [27], it is foreseeable that infants will spend more time on ventilators. Thus, it becomes imperative to incorporate neurodevelopmental interventions in the NICU care of this target population during early postnatal life. Our findings and standardized guidelines of HFJV-KC should encourage readers to implement this team approach to skin-to-skin care in the smallest preterm infants while on high-frequency jet ventilation, thus promoting parental bonding and alleviating the separation paradigm of NICU in our most vulnerable patients.

## Figures and Tables

**Table 1:** Patient characteristics of neonates who received kangaroo care on high-frequency jet ventilator.

Gestational Age (Weeks) ^a^	24 0/7 (23 4/7–24 6/7)
Birth weight (grams) ^a^	670 (603–708)
Female	7/13 (54%)
Weight at KC (grams) ^a^	1034 (868–1180)
Inborn patients (n)	12/13 (92%)
Respiratory Distress Syndrome	13/13 (100%)
Surfactant Administration	13/13 (100%)
DOL to first KC (days) ^a^	16(13–20)
Number of times KC declined/KC offered	122/218 (55.9%)
Number of times KC performed/KC offered	96/218 (44%)
Cumulative duration for KC per patient (hours) ^a^	11 (6–15)

^a^ Median (Interquartile range); KC—kangaroo care.

**Table 2:** Comparison of change in vital signs with performance of KC for 1 h vs. >1 h.

	1 h	>1 h	*p* Value
Δ Heart rate	0.5 [14.9] N = 30	−3.0 [13.2] N = 66	NS
Δ SpO_2_ (%)	1.7 [0.9] N = 30	0.7 [0.6] N = 66	NS
Δ FiO_2_ (%)	−0.1 [0.9] N = 29	−1.5 [0.7] N = 66	NS
Δ Temperature (F)	0.02 [0.1] N = 28	−0.12 [0.1] N = 53	NS

Δ: Post HFJV-KC—Pre HFJV-KC; SpO_2_: Oxygen Saturation; FiO_2_: Oxygen Requirement. Mean [Standard error of mean]; N = number of occurrences of HFJV-KC, NS = not significant.

## Data Availability

The data that support the findings of this study are available from the corresponding authors upon reasonable request.

## Abbreviations

The following abbreviations are used in this manuscript:

KC	Kangaroo Care
HFJV	High-Frequency Jet Ventilation
NICU	Neonatal Intensive Care Unit
HR	Heart rate
SpO_2_	Oxygen saturation
T	Axillary temperature
PEEP	Positive End Expiratory Pressure

## References

[1] Cong X., Cusson R.M., Walsh S., Hussain N., Ludington-Hoe S.M., Zhang D. (2012). Effects of skin-to-skin contact on autonomic pain responses in preterm infants. J. Pain.

[2] Evereklian M., Posmontier B. (2017). The Impact of Kangaroo Care on Premature Infant Weight Gain. J. Pediatr. Nurs..

[3] Ludington-Hoe S.M., Anderson G.C., Swinth J.Y., Thompson C., Hadeed A.J. (2004). Randomized controlled trial of kangaroo care: Cardiorespiratory and thermal effects on healthy preterm infants. Neonatal Netw..

[4] Ludington-Hoe S.M., Johnson M.W., Morgan K., Lewis T., Gutman J., Wilson P.D., Scher M.S. (2006). Neurophysiologic assessment of neonatal sleep organization: Preliminary results of a randomized, controlled trial of skin contact with preterm infants. Pediatrics.

[5] Feldman R., Rosenthal Z., Eidelman A.I. (2014). Maternal-preterm skin-to-skin contact enhances child physiologic organization and cognitive control across the first 10 years of life. Biol. Psychiatry.

[6] Mörelius E., Theodorsson E., Nelson N. (2005). Salivary cortisol and mood and pain profiles during skin-to-skin care for an unselected group of mothers and infants in neonatal intensive care. Pediatrics.

[7] Jayaraman D., Mukhopadhyay K., Bhalla A.K., Dhaliwal L.K. (2017). Randomized Controlled Trial on Effect of Intermittent Early Versus Late Kangaroo Mother Care on Human Milk Feeding in Low-Birth-Weight Neonates. J. Hum. Lact..

[8] WHO (2003). Kangaroo Mother Care: A Practical Guide.

[9] Baley J. (2015). Skin-to-Skin Care for Term and Preterm Infants in the Neonatal ICU. Pediatrics.

[10] Kledzik T. (2005). Holding the very low birth weight infant: Skin-to-skin techniques. Neonatal Netw..

[11] DiMenna L. (2006). Considerations for implementation of a neonatal kangaroo care protocol. Neonatal Netw..

[12] Ludington-Hoe S.M., Ferreira C., Swinth J., Ceccardi J.J. (2003). Safe criteria and procedure for kangaroo care with intubated preterm infants. J. Obstet. Gynecol. Neonatal Nurs..

[13] Engler A.J., Ludington-Hoe S.M., Cusson R.M., Adams R., Bahnsen M., Brumbaugh E., Coates P., Grieb J., McHargue L., Ryan D.L. (2002). Kangaroo care: National survey of practice, knowledge, barriers, and perceptions. MCN Am. J. Matern. Child. Nurs..

[14] Chan G., Bergelson I., Smith E.R., Skotnes T., Wall S. (2017). Barriers and enablers of kangaroo mother care implementation from a health systems perspective: A systematic review. Health Policy Plan..

[15] Varty D., Minhas K., Gillis S., Rourke S. (2023). Skin-to-Skin Therapy on High-Frequency Jet Ventilation: A Trauma-Informed Best Practice. Can. J. Respir. Ther..

[16] Bisanalli S., Nesargi S., Govindu R.M., Rao S.P. (2019). Kangaroo Mother Care in Hospitalized Low Birth-Weight Infants on Respiratory Support: A Feasibility and Safety Study. Adv. Neonatal Care.

[17] Lorenz L., Dawson J.A., Jones H., Jacobs S.E., Cheong J.L., Donath S.M., Davis P.G., Kamlin C.O.F. (2017). Skin-to-skin care in preterm infants receiving respiratory support does not lead to physiological instability. Arch. Dis. Child. Fetal Neonatal Ed..

[18] Heimann K., Vaessen P., Peschgens T., Stanzel S., Wenzl T.G., Orlikowsky T. (2010). Impact of skin to skin care, prone and supine positioning on cardiorespiratory parameters and thermoregulation in premature infants. Neonatology.

[19] Welch M.G., Myers M.M., Grieve P.G., Isler J.R., Fifer W.P., Sahni R., Hofer M.A., Austin J., Ludwig R.J., Stark R.I. (2014). Electroencephalographic activity of preterm infants is increased by Family Nurture Intervention: A randomized controlled trial in the NICU. Clin. Neurophysiol..

[20] Feldman R., Eidelman A.I. (2003). Skin-to-skin contact (Kangaroo Care) accelerates autonomic and neurobehavioural maturation in preterm infants. Dev. Med. Child. Neurol..

[21] Feldman R., Eidelman A.I., Sirota L., Weller A. (2002). Comparison of skin-to-skin (kangaroo) and traditional care: Parenting outcomes and preterm infant development. Pediatrics.

[22] Bohnhorst B., Heyne T., Peter C.S., Poets C.F. (2001). Skin-to-skin (kangaroo) care, respiratory control, and thermoregulation. J. Pediatr..

[23] Bohnhorst B., Gill D., Dördelmann M., Peter C.S., Poets C.F. (2004). Bradycardia and desaturation during skin-to-skin care: No relationship to hyperthermia. J. Pediatr..

[24] Wagaman M.J., Shutack J.G., Moomjian A.S., Schwartz J.G., Shaffer T.H., Fox W.W. (1979). Improved oxygenation and lung compliance with prone positioning of neonates. J. Pediatr..

[25] Martin R.J., Miller M.J., Carlo W.A. (1986). Pathogenesis of apnea in preterm infants. J. Pediatr..

[26] Moore B., Roark T., DePriest D., Wright L., Carpenter A., Willet K., Bhandary P., Giannone P., Hanna M., Patra A. (2019). Provision of Kangaroo Care on High Frequency Jet Ventilator in Extremely Premature Neonates Is Not Associated with Increased Risk of Unplanned Extubation and Severe Intraventricular Hemorrhage.

[27] Stoll B.J., Hansen N.I., Bell E.F., Walsh M.C., Carlo W.A., Shankaran S., Laptook A.R., Sánchez P.J., Van Meurs K.P., Wyckoff M. (2015). Trends in Care Practices, Morbidity, and Mortality of Extremely Preterm Neonates, 1993–2012. JAMA.

